# Dosimetric correlation between exit dose maps and anatomical changes in head and neck cancer treatment on Halcyon‐E: A gamma analysis approach

**DOI:** 10.1002/acm2.70710

**Published:** 2026-07-22

**Authors:** Jayane Julia Pereira da Silva, Caio Weber Mendanha Ribeiro, Jaime Luiz Ludwig, Daniela Rocha Medrado, Bruno Nunes Melo da Silva, Ayane Cristine Alves Sarmento, Edilmar de Moura Santos

**Affiliations:** ^1^ Department of Radiotherapy Liga Contra o Câncer Natal Rio Grande do Norte Brazil; ^2^ Department of Radiotherapy Hospital São Marcos Teresina, Piauí Brazil

**Keywords:** dosimetric correlation, gamma index, head and neck cancer, image‐guided radiotherapy, replanning strategies

## Abstract

**Background:**

Head and neck cancer (HNC) has a high incidence in Brazil. Although Image‐Guided Radiation Therapy (IGRT) improves treatment precision, anatomical changes during the treatment course may compromise dose distribution and require replanning.

**Purpose:**

This study investigated the correlation between exit dose fluence and anatomical volumetric variations during HNC radiotherapy. Subsequently, a decision support methodology was proposed and validated to flag potential dosimetric deviations and guide objective replanning strategies.

**Methods:**

A retrospective study was conducted with 11 patients, totaling 312 fractions treated on a Halcyon‐E linear accelerator. Volumetric variations were derived from cone‐beam computed tomography (CBCT) and exit dose fluence maps from each fraction were compared with the reference from the first treatment day using gamma analysis (γ_(1%/1 mm) to γ_(5%/5 mm); 10% threshold). Correlations between normalized volumetric variation (ΔV) and gamma indices were assessed using Kendall's tau coefficient (*α* = 0.05). A decision support methodology for replanning (γ_(1%/1 mm) < 80% and γ_(2%/2 mm) < 90%) was proposed and retrospectively validated in an independent cohort of 20 patients, comprising 618 treatment fractions.

**Results:**

Strong negative correlations were observed between ΔV and gamma indices, with γ_(1%/1 mm) showing the highest sensitivity (*τ* = −0.83), followed by γ_(2%/2 mm) (*τ* = −0.75). Volumetric variations exceeding 5% were associated with significant dosimetric degradation. The validation correctly identified all replanned patients and detected potential indicators of dosimetric divergence not recognized in routine clinical practice.

**Conclusions:**

The thresholds γ_(1%/1 mm) < 80% and γ_(2%/2 mm) < 90%, combined with ΔV > 5%, proved effective for guiding replanning decisions. The proposed methodology demonstrated high sensitivity for detecting dosimetric deviations, with potential to enhance radiotherapy safety and effectiveness.

## 1 INTRODUCTION

Head and neck cancer (HNC) encompasses a range of malignancies affecting the oral cavity, pharynx, larynx, and nearby structures. With significant incidence rates, HNC remains a prominent public health concern in Brazil, particularly in the Southern and Southeastern regions. According to projections by the National Cancer Institute (INCA) for the 2026–2028 triennium, of the 781 000 new cancer cases estimated annually nationwide, excluding non‐melanoma skin cancer, approximately 391 960 occur in men and 389 090 in women, with HNC representing a substantial proportion of these cases.^1^

Radiotherapy is a mainstay in HNC treatment, bolstered by advancements in Image‐Guided Radiotherapy (IGRT). IGRT allows for precise online and offline correction strategies, enhancing dose accuracy and minimizing exposure to surrounding healthy tissues.^2^, ^3^, ^4^ These improvements are especially critical in HNC due to the anatomical complexity of the head and neck region, the proximity to vital organs, and notable tumor volume reductions observed as early as the second treatment week.^5^

While IGRT provides significant therapeutic benefits through increased dosimetric precision, radiotherapy for HNC is still frequently accompanied by adverse effects such as mucositis, xerostomia, and dysphagia. These complications can significantly impact patients' quality of life and treatment adherence.^5^ Adaptive Radiation Therapy (ART) has shown promise in mitigating these issues by adjusting treatment plans in response to anatomical changes over the course of therapy.^6^ However, implementing ART remains challenging due to the high technological costs and operational demands. In high‐volume centers, integrating adaptive workflows requires significant resource optimization to maintain patient throughput, often limiting its widespread routine application.^6^, ^7^

Anatomical changes, such as tumor shrinkage, can alter dose distribution, potentially compromising treatment efficacy and patient outcomes.^5^, ^8^ When these changes are deemed clinically significant, medical physicists and clinicians evaluate the dosimetric impact to determine the necessity of a new computed tomography (CT) simulation and subsequent replanning. However, the existence of a universal threshold for triggering replanning remains uncertain, as no standardized protocol consistently defines this limit in clinical practice. In routine settings, decisions often rely on clinicians’ experience, combining qualitative assessments of anatomical changes from cone‐beam CT scans (CBCT) with institution‐specific guidelines, such as monitoring tumor volume or patient weight loss.^7^ The absence of consensus on when to interrupt treatment for replanning poses a significant challenge, as establishing a clear ’stop’ criterion is complex due to interpatient variability and the lack of objective, universally accepted dosimetric markers.^7^, ^9^

The frequency with which replanning is required in HNC treatment varies widely, with reported rates between 32% and 70%.^7^ To address this variability, Schaly et al. introduced a decision‐support algorithm using gamma index‐based image comparison to establish clinical criteria for replanning, while Lai et al. correlated IGRT displacement with neck thickness and circumference at various levels, recommending replanning for patients showing marked reductions at the mastoid tip level.^10^, ^11^

Despite the widespread use of gamma analysis for detecting dosimetric deviations,^12^ identifying the optimal timing for replanning remains an ongoing challenge due to the lack of standardized, objective criteria, often forcing clinicians to rely on subjective experience and visual inspection.^13^, ^14^ This study aims to propose a decision support methodology to mitigate the risks of underdosing residual tumors or overdosing healthy tissues, standardize re‐evaluation protocols, and facilitate the routine implementation of personalized strategies even in resource‐constrained settings.

## 2 MATERIALS AND METHODS

### 2.1 Study design, equipment and data

This retrospective cohort study analyzed data from 11 patients treated for HNC using the Halcyon‐E system at the Advanced Oncology Center of the Liga Contra o Câncer in Natal, Rio Grande do Norte, Brazil. The Halcyon system is equipped with a 6 MV flattening filter‐free (FFF) beam, characterized by a nominal depth of maximum dose (dmax) of 1.3 cm and an average energy of 1.3 MeV. The system features a fast gantry rotation speed (4 rotations per minute) and a dual‐layer multi‐leaf collimator (MLC) with 1 cm leaves, offset by 5 mm to provide an effective resolution of 5 mm at the isocenter. The maximum field size is 28 × 28 cm^2^. The study included patients of both sexes and all age groups diagnosed with HNC and treated on the Halcyon‐E. No restrictions were applied regarding cancer stage. The sole exclusion criterion was an interval exceeding 15 days between CT simulation and the initiation of treatment.

The analyses were performed using the Eclipse treatment planning system (v. 15.6), resulting in a dataset of 312 volumetric measurements obtained from daily pre‐treatment CBCT scans. The volume of the ‘Body’ structure was extracted from each CBCT image. To ensure consistency across fractions, special attention was given to standardizing the longitudinal extent (cranial‐caudal limits) of the contour for each patient to avoid volume discrepancies caused by daily variations in the CBCT field of view. Subsequently, all contours were reviewed slice‐by‐slice to ensure accuracy and to manually exclude external elements, such as immobilization devices.

### 2.2 Exit dose map and gamma analysis

**FIGURE 1:**
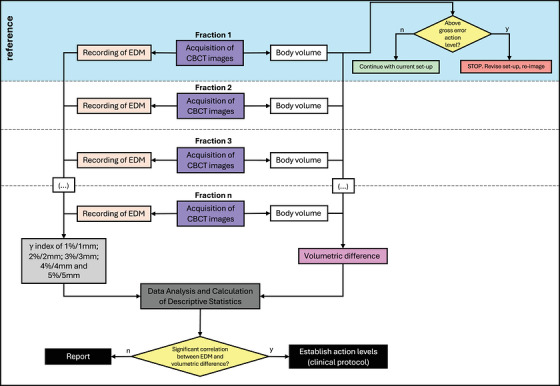
Flowchart of the study methodology. CBCT, Cone‐Beam Computed Tomography; EDM, Exit Dose Map; y, yes; n, no.

Data extraction within Eclipse was performed using the system's “Statistics” tool. The first kVCBCT scan served as the baseline reference for subsequent assessments, as it most closely approximated the anatomy from the initial planning CT. For each delivered treatment fraction, the Digital Megavoltage Imaging (DMI) detector of the Halcyon‐E recorded the Exit Dose Map (EDM), defined as the two‐dimensional distribution of the radiation fluence transmitted through the patient's anatomy during treatment delivery. Image acquisition was performed dynamically during beam‐on time, with standard dark‐field, flood‐field, and pixel defect corrections automatically applied by the Halcyon system to maintain detector uniformity. Daily detector stability was verified through the Machine Performance Check (MPC), which includes dark‐field and open‐field acquisitions. The resulting EDMs were subsequently analyzed using the Portal Dosimetry system. Figure 1 presents a flowchart illustrating the methodology used.

A detailed gamma index analysis was performed for each treatment fraction, comparing the measured fluence distribution for each session to the reference fluence obtained on the first treatment day. This comparison was performed using the composite fluence of the treatment fields, providing a comprehensive evaluation of the overall daily dose distribution transmitted through the patient. Multiple tolerance criteria (γ_(1%/1 mm), γ_(2%/2 mm), γ_(3%/3 mm), γ_(4%/4 mm), and γ_(5%/5 mm)) were applied with a 10% low‐dose threshold. The primary aim of this analysis was to assess cumulative changes throughout the treatment course in terms of dose difference (DD) and distance‐to‐agreement (DTA).^12^ Monitoring these variations enabled the evaluation of the stability and accuracy of the delivered treatment, identifying potential treatment‐related deviations.

### 2.3 Statistical analysis and protocol development (Phase I)

Statistical analysis included specific tests to verify the underlying assumptions for each patient's dataset. The Shapiro–Wilk test was employed to assess the normality of the data distribution, while the Durbin–Watson test verified the independence of residuals—A necessary step to ensure the absence of significant autocorrelation. Homoscedasticity was evaluated using the Breusch‐Pagan test to detect potential non‐constant variance in the residuals. Additionally, the Interquartile Range (IQR) method was applied to robustly identify and manage outliers. Finally, boxplots were utilized to visualize data dispersion, highlighting central tendency measures such as the median and potential outliers, providing a clear and informative overview of the data distribution.

Building upon the statistical verification, an investigation was conducted to explore the relationship between gamma analysis metrics and anatomical volumetric variations. To assess these correlations, Kendall's tau (τ) coefficient was selected. The use of this non‐parametric test was necessitated because the data distributions were anticipated to reject normality, thereby precluding the use of parametric tests like Pearson's correlation. The objective was to identify significant correlations that could characterize the dosimetric impact of anatomical changes observed throughout the treatment.

Leveraging the identified correlations, a decision support methodology was developed to guide clinical decision‐making. Constructed based on the optimal statistical fit, this methodology aimed to establish a practical framework linking volumetric variations, gamma analysis metrics, and relevant clinical factors. The objective was to provide radiation oncologists and medical physicists with a quantitative tool to evaluate the necessity of treatment replanning, thereby optimizing the balance between therapeutic efficacy and patient safety.

Furthermore, specific action thresholds were derived from the study findings. These cutoff values (γ_(1%/1 mm) < 80% and γ_(2%/2 mm) < 90%) were established empirically by cross‐referencing the dosimetric degradation with volumetric changes. The analysis demonstrated a consistent and marked decline in gamma passing rates when ΔV exceeded 5%. These values were therefore established to trigger clinical interventions, ranging from plan review to replanning strategies. Specifically, volumetric deviations or significant shifts in gamma analysis metrics exceeding these thresholds served as indicators for immediate reassessment. This framework was proposed to ensure robust quality control and precision throughout the radiotherapy treatment course.

### 2.4 Retrospective validation (Phase II)

To assess the clinical applicability of the developed methodology, a second phase of the study was conducted. The decision support criteria established in Phase I were retrospectively applied to an independent and expanded cohort of 20 patients, comprising a total of 618 analyzed treatment fractions. This validation phase aimed to determine whether the protocol could accurately identify the need for replanning and detect potential deviations that had not been previously recognized in patients who underwent conventional treatment without intervention.

## 3 RESULTS

### 3.1 Statistical analysis of correlation

The statistical analysis began with assessments of data distribution and assumptions for regression modeling. The Shapiro–Wilk test was applied to evaluate normality for both normalized volume variations (ΔV) and gamma indices across all criteria (γ_(1%/1 mm) to γ_(5%/5 mm)). Normality was rejected for ΔV in all 11 patients (*p* < 0.05) and for the majority of gamma indices (*p* < 0.05), with the exception of the γ_(3%/3 mm), γ_(4%/4 mm), and γ_(5%/5 mm) criteria in Patient 6. Additionally, the Durbin–Watson test confirmed data independence, and the Breusch–Pagan test indicated homoscedasticity in the residuals (*p* > 0.05 for most cases).

Kendall's τ analysis demonstrated consistent negative correlations between ΔV and gamma passing rates across all criteria, indicating that larger ΔV were associated with lower gamma passing rates. The strongest correlation was observed for the most stringent criterion, γ_(1%/1 mm) (*τ* = ‐0.83, *p* < 0.001), followed by γ_(2%/2 mm) (*τ* = ‐0.75, *p* < 0.001). The remaining criteria (3%/3 mm to 5%/5 mm) exhibited moderate negative correlations, with τ values ranging from −0.70 to −0.40. These findings underscore the superior sensitivity of tighter gamma criteria to anatomical deviations, whereas looser tolerances showed weaker, albeit significant, associations.

**FIGURE 2:**
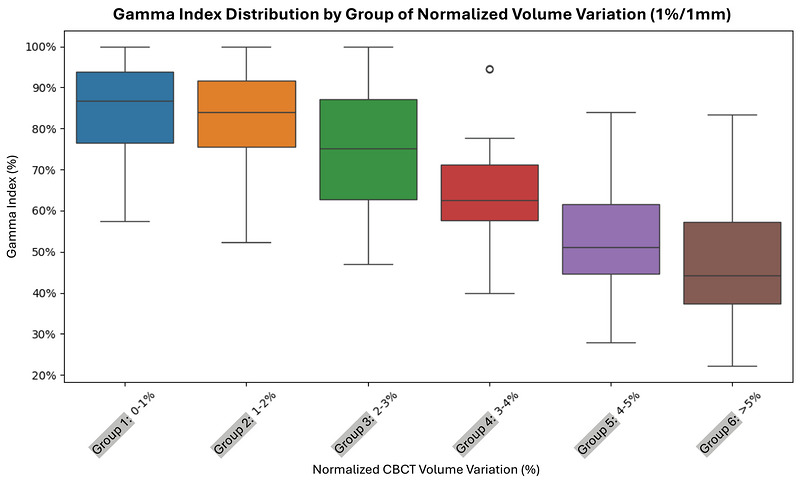
Boxplot distribution of γ_(1%/1 mm) according to binned ΔV groups. Medians decrease progressively with increasing volume change, highlighting greater dosimetric impact in higher ΔV categories.

**FIGURE 3:**
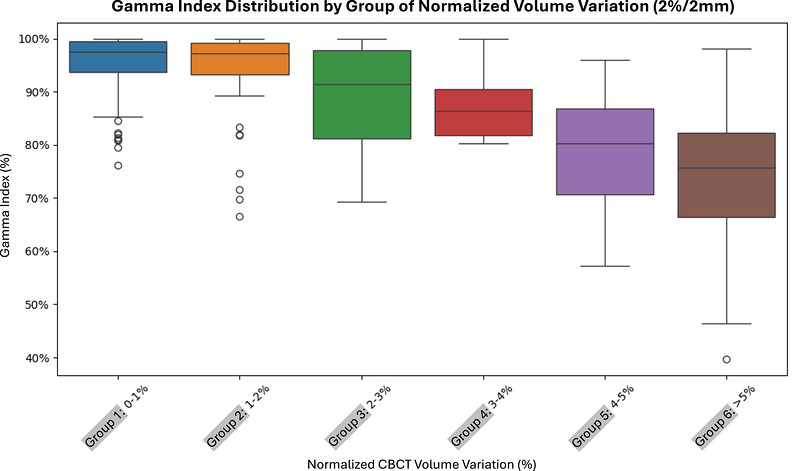
Boxplot distribution of γ_(2%/2 mm) according to binned ΔV groups. Similar declining trend as observed for the stricter criterion, but with overall higher passing rates.

To visualize the relationship between volumetric variations and dosimetric agreement, the data were stratified into discrete ΔV intervals (0%–1%, 1%–2%, 2%–3%, 3%–4%, 4%–5%, and > 5%). Boxplots (Figure 2 for γ_(1%/1 mm); Figure 3 for γ_(2%/2 mm)) were generated to provide a robust statistical assessment of these distribution patterns and highlight distinct sensitivity profiles.

Under the γ_(2%/2 mm) criterion, intervals with volumetric variations of up to 4% maintained high gamma indices, with overall higher passing rates and tighter data distributions. In contrast, under the more stringent γ_(1%/1 mm) criterion, an earlier and more pronounced degradation of dosimetric agreement was observed. A progressive decline in median passing rates was evident, dropping from approximately 87% in the 0%–1% bin to below 50% in the > 5% bin. The expanded interquartile ranges and increased data dispersion in higher ΔV categories further underscore the variability and the prevalence of critical dosimetric deviations associated with substantial anatomical changes. In both scenarios, the plots confirm a clear decreasing trend in pass rates as anatomical volumetric variations increase.

**FIGURE 4:**
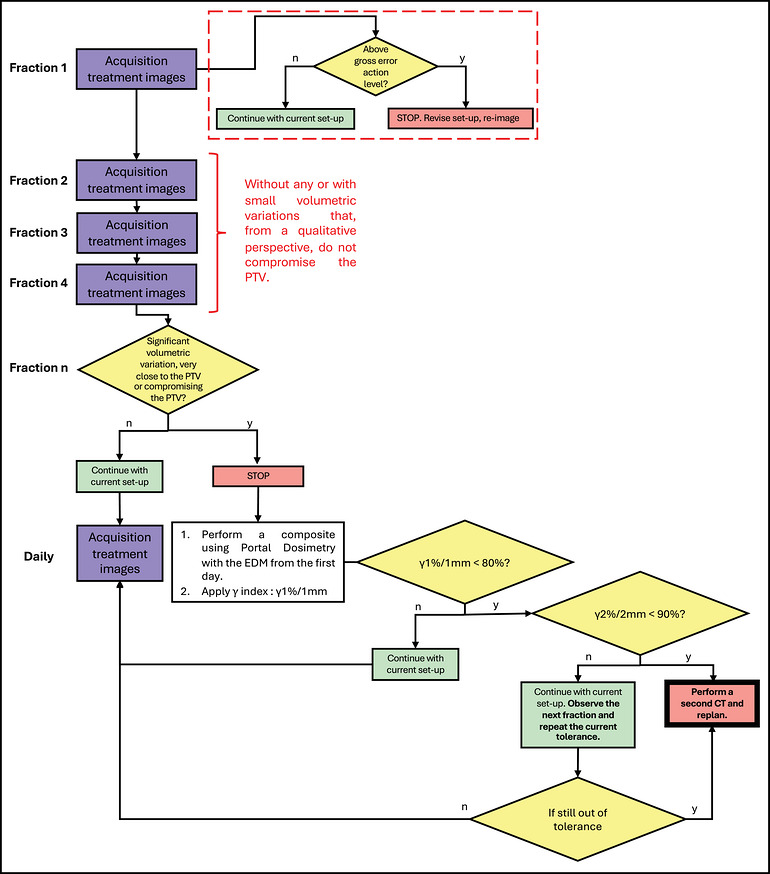
Schematic of the proposed decision support methodology. Primary trigger: γ_(1%/1 mm) < 80%; secondary trigger: γ_(2%/2 mm) < 90%. Thresholds are informed by the observed correlations between volume change and dosimetric agreement.

Collectively, these results demonstrate a robust negative correlation, confirming that anatomical volumetric variations are a critical determinant of dosimetric degradation. This relationship provided the empirical rationale for the decision support methodology (Figure 4), which establishes a γ_(1%/1 mm) passing rate < 80% as the primary trigger and a γ_(2%/2 mm) passing rate < 90% as the secondary trigger to guide replanning decisions.

### 3.2 Retrospective methodology validation

The proposed decision support methodology was retrospectively applied to an expanded cohort of 20 patients with HNC, comprising a total of 618 treatment fractions delivered on a Halcyon‐E linear accelerator. The absence of additional exclusion criteria in this phase was intentional, aiming to evaluate the methodology's performance under conditions that reflect routine clinical practice, which is characterized by substantial interpatient variability. This approach allows assessment of the methodology's robustness and practical applicability while minimizing selection bias and enhancing its external validity.

Daily EDMs were compared with the reference fluence from the first treatment day using the Portal Dosimetry tool. The primary decision support interruption criterion was defined as γ_(1%/1 mm) < 80%, while the secondary criterion, γ_(2%/2 mm) < 90%, was evaluated to confirm the need for acquisition of a new planning CT and, consequently, treatment replanning.

The methodology correctly identified all 6 patients who underwent clinical replanning. In these instances, alerts consistently preceded or coincided with the clinical decision, with the primary criterion triggering first in most cases, followed by confirmation via the secondary criterion. Notably, among the 14 patients who did not undergo replanning, the methodology flagged critical alerts in several cases, indicating undetected dosimetric deviations during routine monitoring.

Table 1 summarizes the specific monitoring results for the validation cohort. To distinguish between the initial dosimetric divergence and its maximum degradation, two key metrics are presented: the “Alert Fraction”, marking the first treatment fraction where the methodology triggered a warning, and the “Minimum Gamma”, representing the lowest passing rate recorded for the original plan throughout the treatment course.

**TABLE 1:** Retrospective application of the decision support methodology. The “Replan.?” column indicates whether a clinical intervention actually occurred. “Alert” denotes the first fraction where the threshold was breached. “Minimum” represents the lowest gamma value observed for the original plan, reflecting the maximum degradation of dosimetric agreement.

Patient	Total fractions	Replan.?	Alert γ1%/1 mm (Fraction)	Mínimum γ1%/1 mm (Fraction)	Alert γ2%/2 mm (Fraction)	Mínimum γ2%/2 mm (Fraction)	Observation / Outcome
P1	35	Yes	79.6% (F. 3)	35.7% (F. 13)	89.9% (F. 5)	51.1% (F. 13)	Index recovery: Indices restored to > 97% following replanning.
P2	33	Yes	79.8% (F. 12)	**23.9% (F. 16)**	57.7% (F. 16)	57.7% (F. 16)	Marked index decline: Replan triggered at F. 20.
P3	33	Yes	75.7% (F. 6)	36.8% (F. 11)	72.9% (F. 11)	70.8% (F. 21)	Indices stabilized post‐replanning.
P4	33	Yes	75.9% (F. 2)	**29.4% (F. 13)**	86.4% (F. 4)	50.7% (F. 13)	Thresholds crossed: Replanning performed at F. 8.
P5	33	Yes	74.5% (F. 4)	48.6% (F. 31)	87.9% (F. 4)	72.2% (F. 31)	Late index decline: Primary threshold crossed at F. 29.
P6	33	Yes	78.1% (F. 4)	32.6% (F. 28)	83.3% (F. 12)	51.0% (F. 25)	Fluctuating indices: Primary threshold crossed repeatedly from F. 12.
P7	30	No	78.5% (F. 15)	78.5% (F. 15)	—	97.9% (F. 15)	Index stability: Secondary criterion remained above tolerance.
P8	33	No	69.3% (F. 13)	57.3% (F. 31)	80.9% (F. 17)	76.5% (F. 31)	Late threshold breach at F. 31.
P9	33	No	78.6% (F. 3)	62.4% (F. 27)	88.9% (F. 13)	84.0% (F. 33)	Indices maintained above secondary threshold.
P10	33	No	70.9% (F. 6)	**18.9% (F. 31)**	79.1% (F. 23)	42.2% (F. 31)	Potential deviation flagged: Dual‐criteria crossed by F. 23.
P11	25	No	78.6% (F. 2)	38.1% (F. 21)	82.2% (F. 3)	57.3% (F. 21)	Potential deviation flagged: Persistent thresholds breach from F. 13.
P12	25	No	79.5% (F. 3)	58.4% (F. 23)	81.3% (F. 4)	77.0% (F. 23)	Thresholds crossed across multiple fractions.
P13	20	No	69.0% (F. 5)	31.0% (F. 15)	70.0% (F. 9)	48.2% (F. 15)	Early index decline: Secondary threshold crossed at F. 9.
P14	33	No	72.5% (F. 4)	43.4% (F. 33)	75.3% (F. 21)	61.5% (F. 33)	Indices maintained above secondary threshold.
P15	29	No	74.9% (F.4)	27.7% (F. 28)	89.3% (F. 10)	47.6% (F. 28)	Potential deviation flagged: Dual‐criteria crossed from F. 10.
P16	25	No	67.2% (F. 2)	56.6% (F. 11)	89.4% (F. 6)	78.5% (F. 11)	Fluctuating indices: Transient drops followed by recovery.
P17	33	No	79.1% (F. 10)	48.2% (F. 32)	89.7% (F. 12)	76.8% (F. 32)	Late threshold breach at F. 28.
P18	33	No	74.6% (F. 10)	33.4% (F. 24)	89.9% (F. 11)	63.4% (F. 24)	Potential deviation flagged: Dual‐criteria crossed by F. 11.
P19	33	No	69.8% (F. 4)	**16.6% (F. 27)**	77.2% (F. 7)	31.1% (F. 30)	Potential deviation flagged: Dual‐criteria crossed by F. 7.
P20	33	No	72.6% (F. 9)	20.4% (F. 22)	88.5% (F. 17)	35.2% (F. 22)	Potential deviation flagged: Dual‐criteria crossed by F. 17.

Within the replanned group, minimum gamma values consistently fell to critical levels (e.g., 23.9% for Patient P2 and 29.4% for Patient P4), quantitatively validating the clinical decision to intervene. In these cases, the alert fraction typically preceded the minimum gamma, demonstrating the methodology's capacity to provide early warnings before maximum degradation occurred.

**FIGURE 5:**
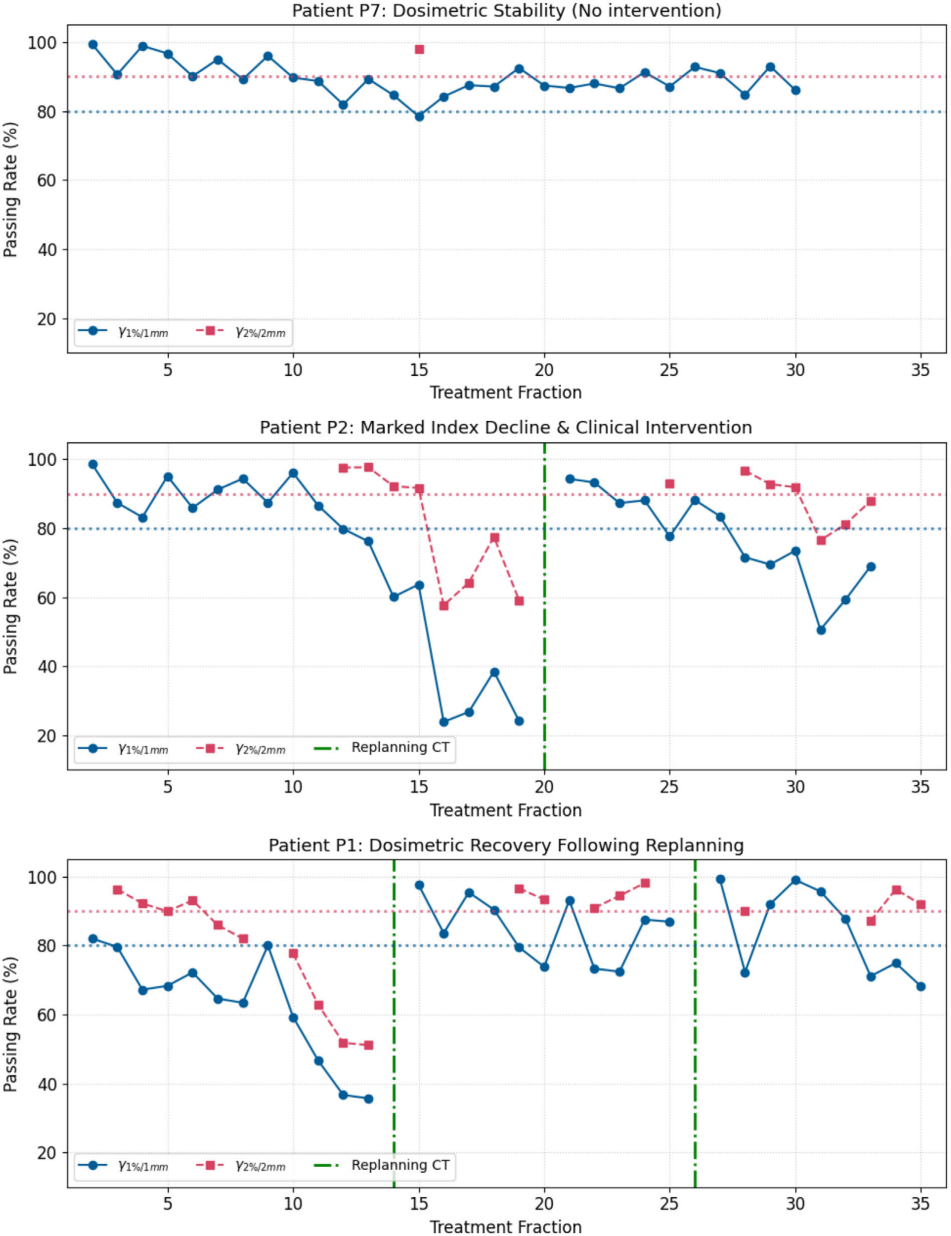
Longitudinal evaluation of γ_(1%/1 mm) (blue circles) and γ_(2%/2 mm) (red squares) passing rates across treatment fractions for three representative patients. Horizontal dotted lines indicate the primary (< 80%) and secondary (< 90%) decision support thresholds. (Top) Patient P7 demonstrates dosimetric stability, consistently remaining above the primary alert threshold. (Middle) Patient P2 exhibits a marked, progressive decline across both criteria, triggering alerts prior to the clinical replanning intervention (green dash‐dotted line). (Bottom) Patient P1 illustrates dosimetric recovery, where indices dropped to critical levels but were restored to > 95% following the implementation of a new planning CT. Missing points in the γ_(2%/2 mm) series represent fractions where the secondary evaluation was not triggered.

To further illustrate the longitudinal behavior of the dosimetric indices and the efficacy of the proposed clinical interventions, Figure 5 presents the fraction‐by‐fraction evolution for three representative clinical scenarios: dosimetric stability, marked index decline requiring intervention, and dosimetric recovery.

Conversely, within the non‐replanned group, the methodology retrospectively identified potential indicators of dosimetric divergence that had not been detected by routine monitoring. Notably, Patients P19 and P10 recorded minimum gamma indices of 16.6% and 18.9% (γ_(1%/1 mm)), respectively. These values represent significant deviations from the planned dose distribution that occurred without triggering clinical alerts during the standard clinical routine.

## 4 DISCUSSION

### 4.1 Dosimetric correlation and action thresholds

The results of this study reinforce previous findings demonstrating a strong relationship between anatomical changes, such as tumor volume reduction or increase, weight loss, and decreases in cervical thickness, among others, and the subsequent dosimetric impact during the course of HNC radiotherapy.^7^, ^9^

The strong negative correlation observed via the τ coefficient for the γ_(1%/1 mm) criterion (*τ* = −0.83) highlights its high sensitivity to anatomical volumetric changes, establishing it as a robust primary indicator. In this study, γ_(1%/1 mm) values below 80% identified 92% of cases requiring clinical intervention, reinforcing its role as an early trigger for replanning. This finding is consistent with the approach proposed by Schaly et al.,^10^ who demonstrated that gamma analysis is capable of detecting clinically significant anatomical alterations that compromise dose conformity. This high sensitivity reflects the ability of the stringent gamma criterion to capture subtle dosimetric discrepancies in anatomically complex regions, where steep dose gradients and the proximity of organs at risk demand high dosimetric conformity. In this context, the γ_(1%/1 mm) criterion acts as an early warning signal for plan degradation, enabling proactive identification of treatment fractions at risk of dosimetric failure.

The γ_(2%/2 mm) criterion (*τ* = −0.75) emerged as a reliable secondary marker. Although less restrictive than the γ_(1%/1 mm) criterion, it remained effective in identifying clinically relevant dosimetric variations. In this study, γ_(2%/2 mm) values below 90% were associated with critical cases in 77% of instances requiring intervention, supporting its role as a confirmatory metric. Notably, alerts associated with this criterion occurred, on average, at the 15th fraction, compared to the 6th fraction for the primary criterion. When used in combination, the dual‐criteria strategy (primary: γ_(1%/1 mm) < 80%; secondary: γ_(2%/2 mm) < 90%) provides a balanced framework that maximizes sensitivity without compromising clinical feasibility. This approach proved particularly effective when ΔV exceeded 5%, a threshold consistently associated with critical gamma degradation in both the initial correlation analysis and the retrospective validation.

### 4.2 Clinical validation and workflow integration

The substantial inter‐patient variability observed in this cohort, evidenced by the rejection of normality in the Shapiro–Wilk test, underscores the limitations of adopting universal thresholds and highlights the need for individualized monitoring strategies. This heterogeneity is consistent with literature reports describing replanning rates ranging from 32% to 70% in HNC patients.^7^ These findings evidence the limitations of routine clinical practices based predominantly on subjective visual inspection of CBCT images^7^, ^13^ reinforcing the need to incorporate objective and quantitative dosimetric indicators to guide clinical decision‐making. As noted in the literature, visual assessment often fails to quantify gradual volumetric reduction until significant dosimetric deviation has already occurred.^14^ Thus, the adoption of objective, quantitative dosimetric indicators is crucial to standardize clinical interventions.

The retrospective application of the proposed decision support methodology to 618 treatment fractions from 20 patients provided robust validation of its clinical relevance. The methodology showed full concordance with clinical decisions, correctly identifying all 6 patients who underwent replanning. Importantly, alert thresholds were reached prior to or at the time of the clinical decision, confirming that objective gamma‐based criteria are capable of anticipating the necessity for replanning more reliably than qualitative assessment alone.

Beyond corroborating existing clinical decisions, the methodology revealed potential indicators of dosimetric variations in patients who were not replanned in conventional clinical practice. As detailed in the results, specific cases exhibited marked reductions in gamma passing rates (e.g., minimum gamma values below 20% γ_(1%/1 mm)), proceeding to treatment completion without any intervention. The fact that routine visual verification failed to trigger alerts for such substantial deviations demonstrates that, in the absence of systematic objective monitoring, patients may be exposed to unintended variations in the delivered dose, potentially jeopardizing the therapeutic ratio between tumor control and normal tissue toxicity.

Conversely, the methodology demonstrated excellent specificity in stable cases. Patient P7 maintained consistently high gamma passing rates throughout 30 fractions, with only transient proximity to the primary threshold and sustained values above 97.9% for the secondary criterion. This result confirms that the proposed thresholds minimize excessive false positives and do not interfere with the clinical workflow when the plan remains robust and anatomical variations are minimal. To quantitatively establish the ‘normal fluctuation’ of the measured dose—encompassing daily patient setup variations, machine output stability, and imaging panel response — the daily γ_(1%/1 mm) values of Patient P7 were analyzed. Across the 29 measured fractions, the passing rates demonstrated a mean of 89.5% with a standard deviation (SD) of 4.7%. This establishes the baseline system noise. Consequently, the primary trigger threshold of < 80% represents a deviation greater than 2σ from the stable mean, mathematically confirming that alerts triggered by the methodology reflect true anatomical variations rather than inherent daily fluctuations of the delivery or imaging systems.

Cases classified as exhibiting “critical recovery” further validated the methodology's clinical utility. In Patient P1, early alerts were triggered as early as the 3rd fraction, followed by progressive degradation that culminated in a critical decline in gamma passing rates by the 13th fraction. After replanning based on a new planning CT, gamma values were immediately restored to levels exceeding 97%. This case clearly illustrates that replanning is the appropriate technical response when anatomical changes render the original plan obsolete, restoring both geometric alignment and dosimetric accuracy.

Overall, the results demonstrate that the proposed methodology effectively distinguishes between stable, unstable, and recoverable treatment scenarios. By integrating the assessment of objective and reproducible gamma thresholds, derived from EDMs readily available on the Halcyon‐E, into the traditional process of subjective visual evaluation, the methodology provides a low‐cost, non‐invasive, and clinically feasible solution for treatment monitoring. The strong negative correlation between volumetric variation and gamma pass rates, together with the methodology's ability to identify replanning events, supports its potential as a strategy to enhance treatment safety, improve plan robustness, and standardize decision‐making in HNC radiotherapy.

### 4.3 Limitations and clinical applicability

Despite the promising results and practical utility, several methodological limitations must be acknowledged. First, the reliance on the global CBCT “Body” volume and two‐dimensional EDMs serves as an indirect surrogate for complex anatomical and dosimetric variations. A global volumetric metric is inherently simplistic and may not fully capture critical regional changes, such as specific organ‐at‐risk displacement, asymmetrical weight loss, or localized tumor shrinkage. Furthermore, while 2D gamma analysis is effective for flagging dosimetric inconsistencies, it does not provide three‐dimensional dosimetric specificity to directly quantify dose changes to specific structures,^12^, ^15^ and cannot replace rigorous dose accumulation utilizing deformable image registration (DIR). Thus, a gamma alert should be interpreted as a decision support trigger for further investigation, requiring CBCT review or the acquisition of a new planning CT for detailed assessment. Additionally, regarding the statistical framework, treating multiple treatment fractions from the same patient as independent observations represents a methodological limitation. While the Durbin‐Watson test was employed to verify the independence of residuals and confirm the absence of significant residual autocorrelation, this cross‐sectional approach does not fully account for the longitudinal, repeated‐measures nature of fractionated radiotherapy. This simplified framework was deliberately adopted to establish generalized empirical thresholds across a large volume of daily fractions, though future studies could employ mixed‐effects modeling to further refine intra‐patient correlations. Advanced platforms, such as the Ethos system, directly address these issues by providing CBCT‐based online adaptation and true dose recalculation.

However, the implementation of such high‐end technologies remains financially and operationally inaccessible for many institutions globally due to significant capital costs, specialized training requirements, and clinical workflow demands.^16^, ^17^ The methodology proposed herein is not intended to compete with or replace these advanced online adaptation systems. Instead, it was developed as a highly accessible, low‐cost decision support tool tailored for real‐world, resource‐constrained environments. By leveraging EPID‐based transit dosimetry—a well‐established and cost‐effective quality assurance tool^18^, ^19^—this framework provides a practical and objective mechanism to flag potential deviations and trigger timely replanning strategies, bridging the gap between subjective visual evaluation and fully automated workflows without adding overhead to the clinical routine.

Finally, this validation was conducted at a single institution, using a limited cohort and a specific platform (Halcyon‐E). The generalizability of this specific workflow to other linear accelerator platforms warrants consideration. The proposed methodology is tightly integrated with the Halcyon system's automated, continuous acquisition of EDM via the DMI detector during beam delivery. Translating this approach to conventional C‐arm linacs, such as Varian TrueBeam, Varian Clinac, or Elekta systems, would require the implementation of alternative EPID acquisition modes (e.g., cine‐EPID or integrated transit dosimetry). These alternatives may lack the same level of seamless automation, potentially requiring additional quality assurance steps and increasing the user workload, which represents a limitation for universal deployment. Although the established thresholds proved robust for this dataset, their generalizability requires additional multicenter validation.

### 4.4 Future directions

Currently, the decision support methodology is undergoing prospective clinical validation at our institution, utilizing the established methodology and decision flowchart. This phase aims to confirm the effectiveness of the proposed thresholds, refine them if necessary, and evaluate their impact on clinical outcomes, toxicity profiles, and resource utilization. Future work will also investigate the integration of additional clinical variables—such as weight loss dynamics and cervical thickness variations^11^—to further personalize decision criteria and optimize workflows in HNC radiotherapy.

## 5 CONCLUSIONS

This study demonstrated a robust negative correlation between ΔV and gamma passing rates, with the γ_(1%/1 mm) criterion showing the strongest association, followed closely by γ_(2%/2 mm). These findings support the implementation of γ_(1%/1 mm) < 80% as a primary alert threshold and γ_(2%/2 mm) < 90% as a secondary confirmatory criterion—particularly when accompanied by volumetric changes exceeding 5%—to guide effective and timely replanning decisions.

The retrospective validation in a cohort of 20 HNC patients confirmed the clinical relevance and practical utility of the proposed decision support methodology. Full concordance was observed with existing clinical replanning decisions, while the methodology also revealed potential indicators of dosimetric variations in a subset of non‐replanned cases that would have remained undetected under routine subjective assessment. In patients who underwent replanning, gamma passing rates were consistently restored to clinically acceptable levels, demonstrating that the acquisition of a new planning CT effectively recovers dosimetric accuracy when the original plan becomes compromised by anatomical changes.

By complementing traditional subjective evaluation with objective and reproducible gamma analysis of daily transmitted dose fluence maps available on the Halcyon‐E, the proposed methodology provides a low‐cost, non‐invasive, and clinically feasible decision support tool for personalized treatment monitoring. This strategy enhances treatment safety by flagging potential plan degradation early, avoiding unwarranted replanning in stable cases, and potentially reducing normal tissue toxicity while preserving tumor control through accurate dose delivery.

## AUTHOR CONTRIBUTIONS

**Jayane Julia Pereira da Silva**: Conceptualization; data curation; formal analysis; investigation; methodology; validation; visualization; and writing. **Caio Weber Mendanha Ribeiro**: Conceptualization; formal analysis; investigation; methodology; validation; project administration; supervision; and writing. **Jaime Luiz Ludwig**: Conceptualization; investigation; methodology; supervision; and writing. **Daniela Rocha Medrado**: Investigation; methodology; and writing. **Bruno Nunes Melo da Silva**: Investigation; methodology; and writing. **Ayane Cristine Alves Sarmento**: Methodology and writing. **Edilmar de Moura Santos**: Writing.

## ETHICAL STATEMENT

This study was approved by the Institutional Research Ethics Committee (Registration number CAAE: 79988424.5.0000.5293).

## CONFLICT OF INTEREST STATEMENT

The authors declare that they have no known competing financial interests or personal relationships that could have appeared to influence the work reported in this paper.

## Data Availability

The data that support the findings of this study are available on request from the corresponding author.
